# The Interplay between RNA Editing Regulator ADAR1 and Immune Environment in Colorectal Cancer

**DOI:** 10.1155/2023/9315027

**Published:** 2023-01-10

**Authors:** Guo-Liang Zheng, Guo-Jun Zhang, Yan Zhao, Zhi-Chao Zheng

**Affiliations:** ^1^Department of Gastric Surgery, Cancer Hospital of China Medical University, Liaoning Cancer Hospital and Institute, No. 44, Xiaoheyan Road, Shenyang 110042, Liaoning, China; ^2^Department of Physiology, College of Basic Medical Science, Shenyang Medical College, 146 North Huanghe Street, Yu Hong, Shenyang 110034, Liaoning, China

## Abstract

An abnormality in the regulation of adenosine deaminase acting on RNA (ADAR) enzymes, which catalyzed adenosine-to-inosine (A-to-I) RNA editing, was closely associated with the highly aggressive biologic behavior and poor prognosis in many malignancies. In the present study, we aimed to investigate the relationship among transcript factors-microRNAs regulatory network, immune environment, and ADAR gene in colorectal carcinoma (CRC). The association among the expression levels of ADAR mRNA and copy number variation, methylation, and mutation status were comprehensively analyzed using cBioPortal, Wanderer, and UALCAN databases in CRC datasets. ADAR-transcript factors (TFs) and ADAR-miRNA regulation networks were constructed by Cistrome Cancer and miRWalk2.0, respectively. The full network and subnetworks for ADAR coexpression genes were constructed using the STRING database and visualized by the MCODE module of the Cytoscape app. The relationship between ADAR mRNA expression and the abundance of infiltrating immune cells in CRC patients was explored by the Tumor Immune Estimation Resource, CIBERSORT, and single-gene gene set enrichment analysis (GSEA). ADAR mRNA was elevated and was a cancer essential gene in CRC. ADAR mRNA and transcripts *P110* were significantly elevated in CRC compared to normal controls. Low-level methylation in the promoter region and high copy number amplification of ADAR were responsible for high levels of ADAR mRNA expression. ADAR coexpression genes were mainly involved in immunoregulation, especially T-lymphocyte activation. Hub genes, including CD2, CD274, and FASLG, were also significantly upregulated in the ADAR-high group compared to the control group. Besides, M1 macrophages were enriched in the ADAR-high group compared to the control group. This study demonstrated that ADAR, a new essential gene, was involved in the immune regulator and was a novel immune treatment target in CRC.

## 1. Introduction

Colorectal carcinoma (CRC) was a highly prevalent malignant cancer of the lower digestive tract and was the third leading cause of cancer-related death worldwide [1]. According to the National Comprehensive Cancer Network (NCCN), the incidence and mortality of malignant tumors have been significantly increasing in China [2] but gradually decreasing in the United States [3], especially CRC. Despite the development of diagnostic and therapeutic modalities, CRC remained extremely difficult to efficient treatment and was poor survival, mainly due to high genetic heterogeneity [4, 5]. Therefore, elucidating the mechanisms underlying heterogeneity was very important for CRC diagnosis and therapy.

Adenosine-to-inosine (A-to-I) RNA editing [6] was an important form of posttranscriptional processing, which alters RNA molecule modification by adenosine deaminases acting on RNA (ADARs) in double-stranded RNA (dsRNA). Of them, ADAR (also known as ADAR1) was a member of the adenosine deaminase family [7], which also includes three ADAR proteins (ADAR, ADARB1, and ADARB2) and two ADAR-related proteins (ADAD1 and ADAD2) according to the HUGO Gene Nomenclature Committee. Among them, ADAR [8] has higher editing efficiency than ADARB1, which only edits one particular nucleotide position in the precursor, whereas ADARB2 has not been shown to be an active enzyme due to the lack of a catalytic domain. Both ADAR1 and ADAR2 were ubiquitously expressed (ADAR2 is most abundant in the brain), whereas ADAR3 expression was restrictively expressed in brain tissue [9].

The dysregulation of RNA editing regulator ADARs has been frequently linked to human cancers including hepatocellular carcinoma [10, 11], esophageal squamous cell carcinoma [12], gastric cancer [13], and breast cancer [14, 15]. Unlike other cancer types, the relationship between RNA editing and CRC mostly focuses on the downstream gene AZIN1 [16–18]. Besides, ADAR was alternatively spliced to generate two isoforms [19], commonly known as the constitutive 110 kDa isoform ADAR1 (P110) and interferon-inducible isoform ADAR (P150), suggesting the close association of catalytic activity of ADAR with immune and inflammatory responses. The gene annotation portal BioGPS [20] has shown high expression of ADAR transcripts in T lymphocytes and other immune cells. These observations suggest the important roles of the regulation of RNA editing in CRC progression and prognosis. However, the abnormal alterations of ADAR, corresponding driving forces, and immune association have not been clearly elucidated in CRC.

Notably, we also constructed ADAR-transcript factors and ADAR-miRNA regulation networks to explore the upstream regulator of ADAR. Many studies showed that the TF and miRNA network might be the potential target of therapy for disease [21–23]. The emergence of high-throughput technologies in recent years has provided a powerful tool to detect an increasing number of genetic and epigenetic alterations in CRC, resulting in breakthroughs in the understanding of the biological characteristics of CRC for preventing, diagnosing, and treating. Driven by the massive accumulated high-throughput dataset, we have investigated the interplay between RNA editing regulator ADAR1 and the immune environment in CRC.

In this study, we systematically analyzed ADAR mRNA expression across multiple CRC datasets to uncover the multiple roles of ADAR in CRC progression and prognosis. Our study showed the steadily elevated expression of the RNA editing regulator ADAR in CRC compared with the normal control. Functional analysis revealed significant involvement of ADAR coexpression genes in tumor immune checkpoints in CRC, thereby inducing a proimmunomodulatory effect. Together, the results of this study demonstrate that ADAR likely has immune regulatory roles and may serve as a novel potential biomarker and target immunotherapy for CRC.

## 2. Materials and Methods

### 2.1. Expression Profile of A to I RNA Editing Regulator ADAR in CRC

The UCSC Xena browser database (The University of California Santa Cruz, https://xena.ucsc.edu/), UALCAN database [24], and Human Protein Atlas (HPA, https://www.proteinatlas.org/) were employed to explore the expression profile (mRNA and protein) of A to I RNA editing regulator ADAR in CRC datasets. Essential gene screening from gene effect scores derived from CRISPR knockout screens published by Broad's Achilles and Sanger's SCORE projects was analyzed by the UALCAN database. Negative scores imply cell growth inhibition and/or death following gene knockout.

### 2.2. Expression Pattern of Typical ADAR Transcripts in CRC

ADAR encodes two distinct splicing isoforms: a constitutive 110 kDa isoform ADAR (*p110*) and an interferon-inducible isoform ADAR (*p150*). UCSC Xena browser (The University of California Santa Cruz, https://xena.ucsc.edu/), a bioinformatics tool used to visualize functional genomics data from multiple sources simultaneously, was applied to assess the expression levels of ADAR transcripts (*p110 and p150*) mRNA in TCGA colorectal cancer based on UCSC Toil RNA seq Recompute Compendium (31) (TCGA and GTEx datasets). ADAR transcript RNA sequencing (RSEM TPM, *n* = 19,131) data were downloaded as log2 (TPM + 0.001) values. The Wilcoxon signed-rank test was performed to determine the difference in the expression of ADAR-p150 and ADAR-p110 isoform between CRC and para-cancerous with the help of GraphPad Prism software 7 (San Diego, CA, USA), and *P* < 0.05 was considered statistically significant.

### 2.3. Exploring the Association between Genetic Alteration and mRNA Expression of ADAR Gene in CRC

The mutation status, methylation status, and copy number variation (CNV) of ADAR genes were analyzed with Wanderer [25], UALCAN [24], and cBioPortal databases [26] to elucidate the molecular mechanism of upregulated ADAR at the DNA level. Wanderer [25], an interactive viewer to explore DNA methylation and gene expression data, was first used to explore the differences in ADAR gene-wide methylation status in CRC based on TCGA cancer dataset. The correlation between ADAR gene amplification, methylation, and gene expression data in TCGA CRC datasets was determined by the cBioPortal for Cancer Genomics Portal (https://cbioportal.org).

### 2.4. Construction of ADAR-Transcript Factors and ADAR-miRNA Regulation Networks

ADAR-related transcription factors (TFs) and miRNA networks were developed to identify upregulated ADAR at both the transcriptional and posttranscriptional levels. ADAR-related TFs were retrieved from Cistrome Cancer [27] (https://cistrome.org/CistromeCancer/), a comprehensive resource for predicting TF targets in cancers based on ChIP-seq data. The correlation between TFs and the ADAR mRNA expression level was subsequently estimated with gene expression profiling interactive analysis [28] (https://gepia.cancer-pku.cn; Pearson's *r* > 0.40 and *P* < 0.01). Furthermore, ADAR-related miRNAs were downloaded from the miRWalk2.0 [29] database (https://zmf.umm.uni-heidelberg.de/apps/zmf/mirwalk2/). ADAR-TFs and ADAR-miRNA pairs were visualized using Cytoscape V3.6.1 software [30].

### 2.5. Construction of ADAR mRNA Coexpression Network in CRC

Genes coexpressed with ADAR were identified using the cBioPortal for Cancer Genomics (https://www.cbioportal.org/) with multidimensional cancer genomics datasets. Spearman's correlation coefficient >0.4 and *P* < 0.01 were set as the cut-off criteria. The protein-protein interaction (PPI) network was constructed with the Search Tool for the Retrieval of Interacting Genes Database (STRING) [31](https://www.string-db.org/), and a confidence score >0.4 was considered significant. The hub modules of the PPI network were determined using the molecular complex detection (MCODE) module in Cytoscape with cut-off criteria: degree = 15, node score = 0.2, k-core = 2, and max depth = 100. ADAR mRNA coexpression network and subnetwork were visualized by Cytoscape software.

### 2.6. Identifying Association between Hub Gene Expression Pattern and ADAR in the PPI Subnetwork

Most connectivity modules with ADAR were selected as the hub subnetwork to analyze the expression profile under ADAR high and low expression. ADAR genes were then split in TCGA dataset into low and high expression using the median value of expression profiles in the genome-wide scale as a cut-off value. *P* < 0.05 was considered statistically significant.

### 2.7. GO/KEGG Functional Annotation and Transcript Factor Enrichment Analysis

The genes participating in the coexpression network were uploaded to the Metascape platform [32] (https://metascape.org/) for Gene Ontology (GO) (https://www.geneontology.org/) enrichment analysis, including the cellular component (CC), molecular function (MF), and biological process (BP), the Kyoto Encyclopedia of Genes and Genomes (KEGG) (https://www.genome.jp/kegg/) pathway, and TRRUST transcript factor enrichment analysis. Adjusted *P* < 0.05 using the Benjamini–Hochberg procedure (false discovery rate (FDR)) was considered statistically significant.

### 2.8. Correlation between ADAR Expression and Infiltrating Immune Cells

The correlations between ADAR expression and the abundance of six types of infiltrating immune cells (B cells, CD4^+^ T cells, CD8^+^ T cells, neutrophils, macrophages, and dendritic cells) in CRC were estimated with the partial correlation coefficient (tumor purity) by Tumor IMmune Estimation Resource [33] (TIMER, https://cistrome.shinyapps.io/timer/). Furthermore, we used the CIBERSORT deconvolution algorithm [34] (https://cibersort.stanford.edu/) to estimate the abundance of 22 immune cell types under ADAR high and low expression and to evaluate the corresponding intratumoral immune cell composition. Besides, the single-gene GSEA strategy was utilized to detect the ADAR-related biological pathways (50 cancer hallmark pathways) [35] in the pan-cancer dataset.

## 3. Results

### 3.1. The ADAR mRNA Was Elevated and Was a Cancer Essential Gene in CRC

**Figure 1:**
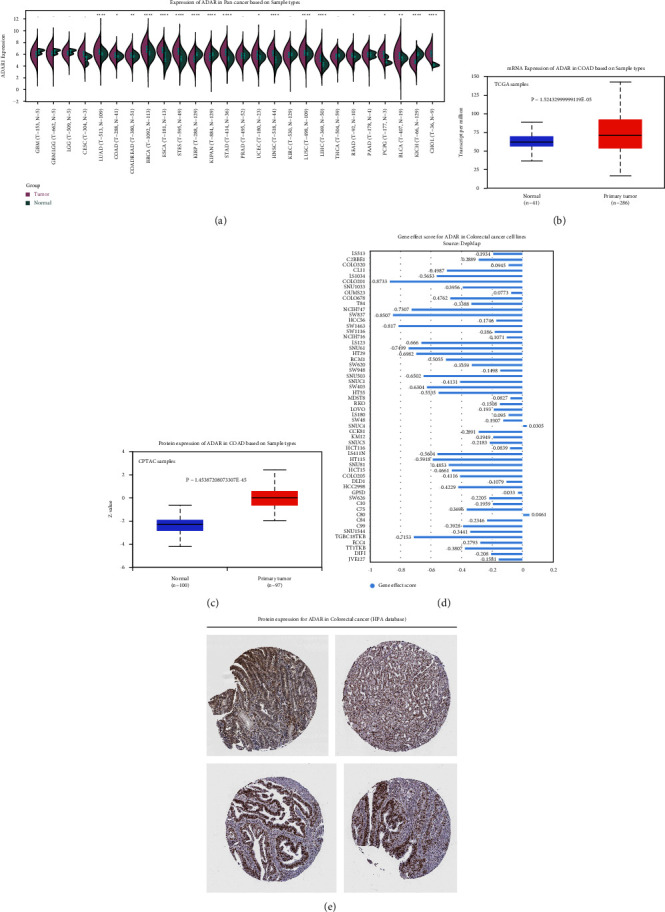
ADAR mRNA was elevated and was a cancer essential gene in CRC. (a) The mRNA expression level of ADAR in TCGA pan-cancer. (b) The mRNA expression level of ADAR in TCGA COAD dataset. (c) The protein expression levels of ADAR in the CPTAC COAD dataset. (d) The gene effect score of ADAR in the COAD cell line from the DepMap database. (e) The protein expression of ADAR in Human Protein Atlas. *P* < 0.05 was considered statistically significant.

The finding from the expression levels of ADAR mRNA showed statistically significant upregulated expressions in pan-cancer databases compared with control (*P* < 0.0001; Figure 1(a)), especially CRC (Figures 1(b), 1(c), and 1(e)), which was extracted from TCGA CRC dataset, CPTAC protein, and Human Protein Atlas (HPA) databases. Interestingly, we found that ADAR was a cancer essential gene in CRC, according to gene effect scores derived from CRISPR knockout screens published by Broad's Achilles and Sanger's SCORE projects (Figure 1(d)). This finding inspires us to further study the ADAR.

### 3.2. The ADAR Transcript p110 Was the Main Regulator of A to I RNA Editing Events in CRC

**Figure 2:**
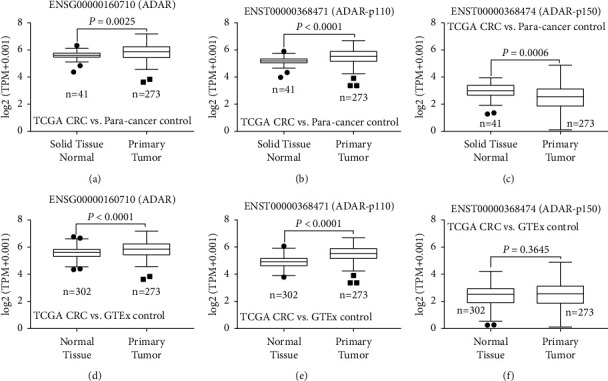
The ADAR transcript p110 was the main regulator of A to I RNA editing events in CRC. (a–c) The levels of ADAR and its classic transcripts (ADAR*-p150* and ADAR*-p110*) between TCGA CRC and paracancer. (d–f) The levels of ADAR and its classic transcripts between TCGA colorectal cancer and GTEx normal colorectal tissues (solid normal tissues). ADAR-*p150* and ADAR-*p110* expression data and curated clinical data were downloaded from the UCSC Xena browser (UCSC toil RNA-seq recompute) to calculate differences in ADAR-*p150* and ADAR-*p110* expression with GraphPad Prism software 7. *P* < 0.05 was considered statistically significant.

As shown in Figures 2(a), 2(b), 2(d), and 2(e), ADAR and ADAR-p110 mRNA were highly expressed in CRC tissues compared with paracancerous tissues. However, ADAR-p150 mRNA transcript was downregulated in CRC tissues in contrast to normal colorectal tissues (Figures 2(c) and 2(f)). These results suggest that the ADAR transcript p110 was the main regulator of A to I RNA editing events in CRC and was involved in multiple biological functions rather than p150.

### 3.3. The Association between ADAR Genetic Alterations and ADAR mRNA in CRC

**Figure 3:**
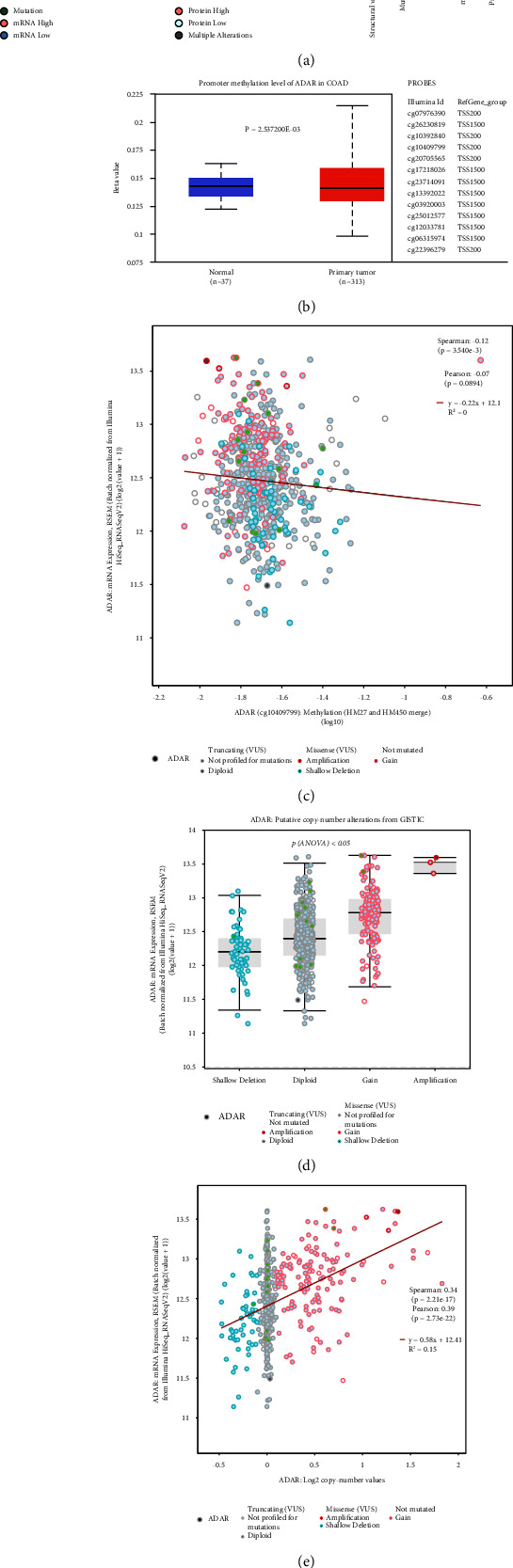
The association between ADAR genetic alterations and ADAR mRNA in CRC. (a) The genetic alteration landscape of ADAR in CRC. (b) ADAR methylation between CRC and paracancer tissues. (c) The correlation between ADAR methylation and mRNA expression. (d) Analysis of the correlation between the expression of ADAR and the copy number. Putative copy-number calls determined using GISITIC 2.0. value: −2 = homozygous deletion; −1 = hemizygous deletion; 0 = neural/no change; 1 = gain; 2 = high level amplification. (e) The correlation between the ADAR copy number and mRNA expression. *P* < 0.05 was considered statistically significant.

As was illustrated in Figure 3(a), the ADAR high copy number was the most significant genomic hallmark. As was shown in Supplementary Figure 1, higher DNA methylation in the ADAR gene and 3′-UTR was observed in both CRC and normal controls. Statistically significant differences in ADAR gene promoter regions were observed, and the ADAR gene was found to be methylated at very low levels in both CRC and normal control tissues (Figures 3(b) and 3(c)). DNA methylation was negatively correlated with the expression level of ADAR (Figure 3(c)). The results suggest that the elevated ADAR gene transcription did not result from methylation, at least not methylation of the promoter region. In addition, the ADAR gene copy number was significantly higher in CRC tissues compared with normal control tissues (Figures 3(d) and 3(e)), suggesting the association of the ADAR mRNA level with the copy number of deep deletions, shallow deletion, diploid, gain, and amplification. There was a significant positive correlation between the DNA copy number and the expression level (Figure 3(e)). In conclusion, high copy number amplification of DNA was the driving force for the increase in the expression level of ADAR.

### 3.4. Multidimensional Regulatory Network Analysis of ADAR-TFs and ADAR-miRNA

**Figure 4:**
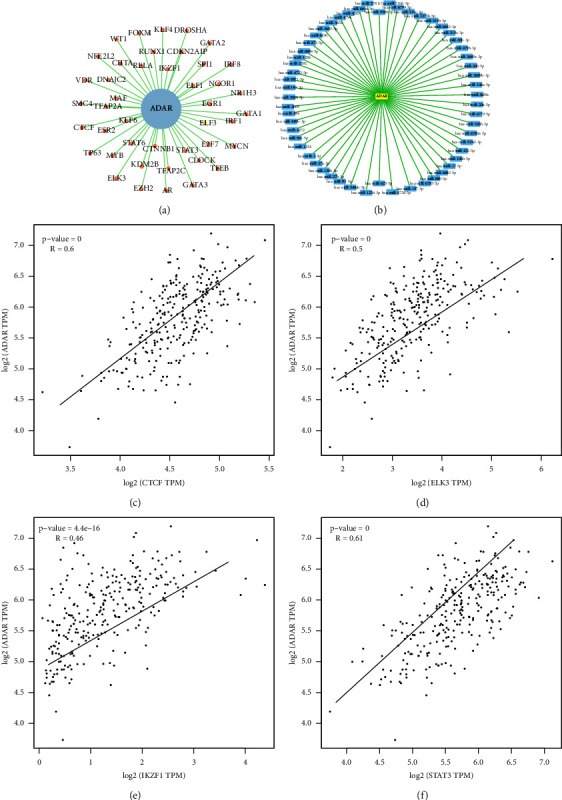
Multidimensional regulatory network analysis of ADAR-TFs and ADAR-miRNAs. (a) The ADAR-TFs pair was extracted from the Cistrome project. (b) The ADAR-miRNA pair was retrieved by miRWalk2.0. (c–f) Correlation curves of TFs highly related to ADAR expression by GEPIA. Pearson *r* > 0.4 and *P* < 0.01 were statistically significant.

In total, 44 ADAR-related TFs were generated based on the Cistrome Cancer web resource at the transcriptional and posttranscriptional regulation levels including *IRF8, STAT6, IRF1, EGR1, MYC, MYCN, KDM2B, EZH2, IKZF1, E2F7, DROSHA, VDR, SPI1, STAT3, MAF, DNAJC2, TP63, MYB, TFAP2C, KLF6, GATA1, ELK3, KLF4, CDKN2AIP, NFE2L2, ELF3, GATA3, NCOR1, CLOCK, TFAP2A, SMC4, FOXM1, CIITA, ESR2, AR, TFEB, CTNNB1, NR1H3, RELA, RUNX1, GATA2, ELF1, WT1,* and *CTCF* (Figure 4(a)). Furthermore, 53 ADAR mRNA-related miRNAs were generated from the miRWalk2.0 database (Figure 4(b)). Of them, ADAR was positively correlated with *CTCF (0.60), STAT3 (0.61), ELK3 (0.50), and IKZF1* (0.46) (Figures 4(c)–4(f)). These findings suggested that ADAR gene transcription regulation was significantly regulated by many transcription factors and microRNAs.

### 3.5. ADAR Coexpression Network, Hub-Network, and Functional Enrichment

**Figure 5:**
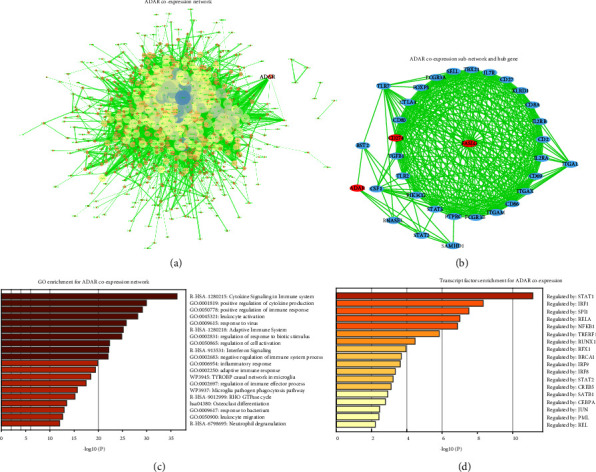
ADAR coexpression network, hub network, and functional enrichment. (a) PPI network construction from *ADAR*-related coexpression analysis. (b) PPI network construction from ADAR-related subnetwork. (c) Functional enrichment analysis of the full network. (d) Transcript factor functional enrichment analysis of the full network. *P* < 0.05 was considered statistically significant.

In total, 1153 positive genes associated with ADAR mRNA expression were obtained through cBioPortal for Cancer Genomics to construct an ADAR-related coexpression gene network. As shown in Figure 5(a), 961 proteins with 8543 nodes were involved in the ADAR-related coexpression network based on the STRING database. A set of 32 proteins that interacted with at least 15 other proteins (*P* < 0.05, FDR <0.05) were selected in the hub network of the PPI network (Figure 5(b)).

Functional enrichment analysis showed that the genes were mainly enriched in BP of lymphocyte activation, cytokine production, inflammatory response, adaptive immune response, leukocyte migration, response to interferon-gamma, and positive regulation of immune response (Figure 5(c)). As shown in Figure 5(d), the results from the transcript factor analysis suggested that ADAR coexpression genes were significantly regulated by STAT1, IRF1, IRF9, and NFKB1. As we know, these transcription factors were closely related to immune regulation, which further suggests that ADAR was significantly involved in the immune regulation process of CRC.

### 3.6. Hub-Subnetwork Derived from the ADAR Coexpression Network and Expression Profile under the ADAR High/Low Group

**Figure 6:**
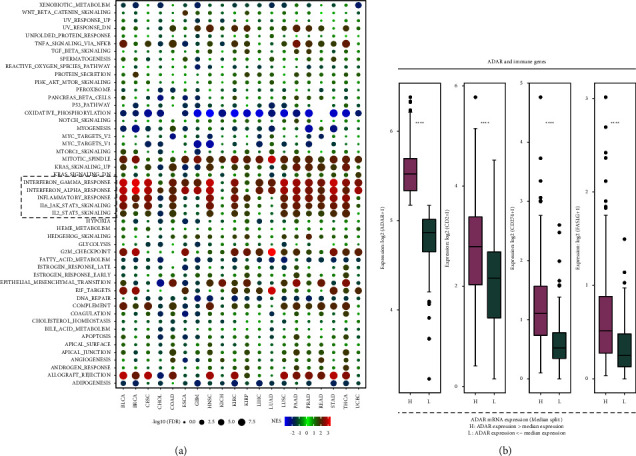
Hub-subnetwork derived from the ADAR coexpression network and expression profile under the ADAR high/low group. (a) Single GSEA (gene set enrichment analysis) for the pan-cancer dataset. (b) Hub gene expression profile difference between ADAR high and low groups. ADAR genes were then split in TCGA dataset into low and high expression using the median value of expression profiles in the genome-wide scale as cut-off.

Finding from single-gene GSEA of ADAR in pan-cancer further confirmed that ADAR participates in the immune signal pathway, especially the interferon pathway (Figure 6(a)). CD2, CD274, and FASLG mRNA levels were significantly higher expressed in the ADAR high group than in the low group (Figure 6(b)). Therefore, ADAR may exert immune regulation function through these immune-related proteins.

### 3.7. The Association between ADAR Gene Expression and Immune Cell Infiltration

**Figure 7:**
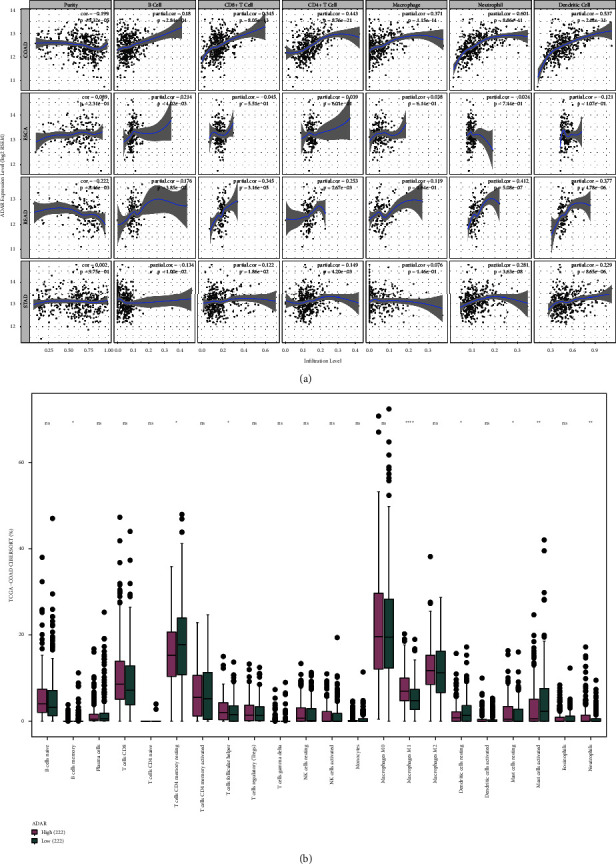
The association between ADAR gene expression and immune cell infiltration. (a) The relationships of ADAR mRNA expression with the abundance of infiltrating immune cells (B cells, CD4+ T cells, CD8+ T cells, neutrophils, macrophages, and dendritic cells) in CRC patients by TIMER for common gastrointestinal tumors. (b) The proportion of immune cells was estimated using CIBERSORT and the LM22 feature matrix. *P* < 0.05 was considered statistically significant.

The correlations of ADAR with infiltrating immune cells (tumor purity) including B cells, CD4^+^ T cells, CD8^+^ T cells, neutrophils, macrophages, and dendritic cells in the gastrointestinal tumors were analyzed by TIMER. As shown in Figure 7(a), ADAR gene expression was closely correlated with dendritic cells, neutrophils, CD4^+^ T cells, macrophages, and CD8^+^ T cells in CRC, especially macrophages. Similar to the previous results (Figure 7(b)), M1 macrophages were enriched in the ADAR high group more than the low group. The abovementioned results in the article suggest that ADAR significantly affects the immune regulation of M1 macrophages.

## 4. Discussion

Immune therapy has been increasingly applied in many cancers that are now characterized by their unique genomic alterations with new diagnostics. Understanding the immune environment in CRC would not only increase the therapeutic efficacy but would also provide a better treatment strategy.

Previous studies have shown a significant correlation between increased ADAR expression and poorer survival outcomes in esophageal squamous cell carcinoma [12, 36] and human hepatocellular carcinoma [10]. In this study, we demonstrated that total ADAR mRNAs and typical transcripts (*p110*) were highly expressed in CRC compared with adjacent paracancerous tissues. However, the p150 mRNA expression level was downregulated between CRC and normal control tissues; ADAR1-*p150* was induced by interferon, whereas ADAR1-p110 was constitutively and ubiquitously expressed.

Finding from genetic alteration, the TF-ADAR-microRNA network showed that high expression of ADAR in CRC might be caused by hypermethylation of the ADAR gene body region, copy number amplification, positive transcription factor, and negative miRNA.

There are few studies on the transcriptional regulation of the RNA editing enzyme ADAR itself, most of which focus on the activity of the ADAR editing enzyme and downstream molecules. From the perspective of transcriptional regulation, our study explored the functional regulation of editing enzymes ADAR with the help of two networks (ADAR-TFs and ADAR-miRNA network's roles). A classic example of transcription factors regulating ADAR, AR served as a transcriptional activator of the ADAR1 promoter to promote HCC tumor growth both in vitro and in vivo [37].

The infiltrated immune cells in the tumor microenvironment played a crucial function in the occurrence and development of tumors. ADAR-related coexpression network and functional enrichment analysis showed that the ADAR coexpressed proteins were mainly enriched in immunoregulation, especially T-lymphocyte activation and cytokine production. The evaluation of immune cell infiltration in CRC using the TIMER database revealed strong correlations between ADAR and tumor purity in CRC. The expression level of ADAR was closely associated with levels of CD8^+^ cell, CD4^+^ cell, neutrophil, and dendritic cell infiltration in CRC. The correlation between ADRA expression and immune cell marker genes suggests that ADRA regulates CRC immunity through multiple immune cell populations by A-to-I dsRNA editing enzyme activity. Studies have shown the involvement of ADAR in inducing an immunomodulatory effect [38]. Our results are consistent with such reports, and these discoveries suggest that ADAR plays an important role in recruiting and governing immune responses in CRC.

Studies have revealed that CRC is different clinically, pathologically, and genetically and could predispose to different clinical assumptions regarding tumorigenesis as well as survival. Resistance to targeted drugs has become an indisputable fact, thereby resulting ineffectiveness of the drug treatment. However, the precise mechanism mediating drug resistance has not been fully understood. The understanding of the molecular and biochemical mechanisms of drug resistance will facilitate the discovery of alternative target drugs. Our study revealed several key genes with high degrees involved in the subnetwork, including CD274, CD20, and FASLG that have been widely used in clinical tumor treatment, and they were positively associated with poor prognosis in CRC. Loss of ADAR1 was demonstrated to defeat resistance to PD-1 checkpoint blockade caused by inactivation of antigen presentation by tumor cells [39], thereby limiting the effective antitumor immunity. The findings provide a new direction for overcoming immunotherapy resistance by regulating ADAR expression.

Although our results are encouraging, there are some limitations to the study: (1) our study was a pure bioinformatics analysis based on TCGA database and online dataset, and further biological experiments were needed to validate our results. (2) It seems to be a contradictory phenomenon that ADAR is both an oncogene and immune-active gene from the perspective of the expression level and immune association, and the follow-up experiments will focus on the role and function of ADAR in CRC.

In conclusion, ADAR expression was closely correlated with multiple immune markers in CRC. The correlations between ADAR and the prognosis and immune cell infiltration provide a foundation for further research on its immunomodulatory role in CRC diagnosis and treatment.

## Data Availability

All the transcriptome sequencing data were obtained from the UCSC Xena database (https://xenabrowser.net/datapages/), the mutational data were obtained from cBioPortal database (http://www.cbioportal.org/), other data that support the findings of this study are available from the corresponding author upon reasonable request.

## Supplementary Materials

Supplementary Materials Supplementary Figure 1: ADAR gene-wide methylation based on Illumina Human Methylation 450 Bead Chip. (A) The Illumina 450K Methylation BeadChip methylation probes the distribution of the ADAR gene in solid normal tissues. (B) The Illumina 450K Methylation BeadChip methylation probes distribution of ADAR gene methylation in CRC tumor tissues. (C) The differential ADAR methylation probe between CRC and solid normal tissues. *P* < 0.05 was considered statistically significant. Data were obtained from the TCGA Wanderer database.

## References

[1] Bray F., Ferlay J., Soerjomataram I., Siegel R. L., Torre L. A., Jemal A. (2018). Global cancer statistics 2018: GLOBOCAN estimates of incidence and mortality worldwide for 36 cancers in 185 countries. *CA: A Cancer Journal for Clinicians*.

[2] Chen W., Zheng R., Baade P. D. (2016). Cancer statistics in China, 2015. *CA: A Cancer Journal for Clinicians*.

[3] Heymach J., Krilov L., Alberg A. (2018). Clinical cancer advances 2018: annual report on progress against cancer from the American society of clinical oncology. *Journal of Clinical Oncology*.

[4] Marisa L., de Reyniès A., Duval A. (2013). Gene expression classification of colon cancer into molecular subtypes: characterization, validation, and prognostic value. *PLoS Medicine*.

[5] Wang X., Fang H., Cheng Y. (2018). The molecular landscape of synchronous colorectal cancer reveals genetic heterogeneity. *Carcinogenesis*.

[6] Gabay O., Shoshan Y., Kopel E. (2022). Landscape of adenosine-to-inosine RNA recoding across human tissues. *Nature Communications*.

[7] Kim U., Wang Y., Sanford T., Zeng Y., Nishikura K. (1994). Molecular cloning of cDNA for double-stranded RNA adenosine deaminase, a candidate enzyme for nuclear RNA editing. *Proceedings of the National Academy of Sciences of the U S A*.

[8] Savva Y. A., Rieder L. E., Reenan R. A. (2012). The ADAR protein family. *Genome Biology*.

[9] Jin Y., Zhang W., Li Q. (2009). Origins and evolution of ADAR-mediated RNA editing. *IUBMB Life*.

[10] Paz N., Levanon E. Y., Amariglio N. (2007). Altered adenosine-to-inosine RNA editing in human cancer. *Genome Research*.

[11] Chan T. H. M., Lin C. H., Qi L. (2014). A disrupted RNA editing balance mediated by ADARs (Adenosine DeAminases that act on RNA) in human hepatocellular carcinoma. *Gut*.

[12] Qin Y. R., Qiao J. J., Chan T. H. M. (2014). Adenosine-to-inosine RNA editing mediated by ADARs in esophageal squamous cell carcinoma. *Cancer Research*.

[13] Okugawa Y., Toiyama Y., Shigeyasu K. (2018). Enhanced AZIN1 RNA editing and overexpression of its regulatory enzyme ADAR1 are important prognostic biomarkers in gastric cancer. *Journal of Translational Medicine*.

[14] Nakano M., Fukami T., Gotoh S., Nakajima M. (2017). A-to-I RNA editing up-regulates human dihydrofolate reductase in breast cancer. *Journal of Biological Chemistry*.

[15] Li X., Sun G., Wu L. (2021). Upregulation of ADAR promotes breast cancer progression and serves as a potential therapeutic target. *Journal of Oncology*.

[16] Lee S. H., Kim H. P., Kang J. K., Song S. H., Han S. W., Kim T. Y. (2017). Identification of diverse adenosine-to-inosine RNA editing subtypes in colorectal cancer. *Cancer Res Treat*.

[17] Wang C., Zou J., Ma X., Wang E., Peng G. (2017). Mechanisms and implications of ADAR-mediated RNA editing in cancer. *Cancer Letters*.

[18] Shigeyasu K., Okugawa Y., Toden S. (2018). AZIN1 RNA editing confers cancer stemness and enhances oncogenic potential in colorectal cancer. *JCI Insight*.

[19] Patterson J. B., Samuel C. E. (1995). Expression and regulation by interferon of a double-stranded-RNA-specific adenosine deaminase from human cells: evidence for two forms of the deaminase. *Molecular and Cellular Biology*.

[20] Wu C., Jin X., Tsueng G., Afrasiabi C., Su A. I. (2016). BioGPS: building your own mash-up of gene annotations and expression profiles. *Nucleic Acids Research*.

[21] Gao M., Zhang Z., Sun J., Li B., Li Y. (2022). The roles of circRNA-miRNA-mRNA networks in the development and treatment of osteoporosis. *Frontiers in Endocrinology*.

[22] Wang Y., Hou J., He D. (2016). The emerging function and mechanism of ceRNAs in cancer. *Trends in Genetics*.

[23] Karreth F. A., Pandolfi P. P. (2013). ceRNA cross-talk in cancer: when ce-bling rivalries go awry. *Cancer Discovery*.

[24] Chandrashekar D. S., Bashel B., Balasubramanya S. A. H. (2017). UALCAN: a portal for facilitating tumor subgroup gene expression and survival analyses. *Neoplasia*.

[25] Díez-Villanueva A., Mallona I., Peinado M. A. (2015). Wanderer, an interactive viewer to explore DNA methylation and gene expression data in human cancer. *Epigenetics and Chromatin*.

[26] Gao J., Aksoy B. A., Dogrusoz U. (2013). Integrative analysis of complex cancer genomics and clinical profiles using the cBioPortal. *Science Signaling*.

[27] Mei S., Meyer C. A., Zheng R. (2017). Cistrome cancer: a web resource for integrative gene regulation modeling in cancer. *Cancer Research*.

[28] Tang Z., Li C., Kang B., Gao G., Li C., Zhang Z. (2017). GEPIA: a web server for cancer and normal gene expression profiling and interactive analyses. *Nucleic Acids Research*.

[29] Dweep H., Gretz N. (2015). miRWalk2.0: a comprehensive atlas of microRNA-target interactions. *Nature Methods*.

[30] Shannon P., Markiel A., Ozier O. (2003). Cytoscape: a software environment for integrated models of biomolecular interaction networks. *Genome Research*.

[31] Szklarczyk D., Gable A. L., Nastou K. C. (2021). The STRING database in 2021: customizable protein-protein networks, and functional characterization of user-uploaded gene/measurement sets. *Nucleic Acids Research*.

[32] Zhou Y., Zhou B., Pache L. (2019). Metascape provides a biologist-oriented resource for the analysis of systems-level datasets. *Nature Communications*.

[33] Li T., Fan J., Wang B. (2017). TIMER: a web server for comprehensive analysis of tumor-infiltrating immune cells. *Cancer Research*.

[34] Newman A. M., Steen C. B., Liu C. L. (2019). Determining cell type abundance and expression from bulk tissues with digital cytometry. *Nature Biotechnology*.

[35] Liberzon A., Birger C., Thorvaldsdóttir H., Ghandi M., Mesirov J. P., Tamayo P. (2015). The molecular signatures database hallmark gene set collection. *Cell Systems*.

[36] Huo X. X., Wang S. J., Song H. (2021). Roles of major RNA adenosine modifications in head and neck squamous cell carcinoma. *Frontiers in Pharmacology*.

[37] Shi L., Yan P., Liang Y. (2017). Circular RNA expression is suppressed by androgen receptor (AR)-regulated adenosine deaminase that acts on RNA (ADAR1) in human hepatocellular carcinoma. *Cell Death and Disease*.

[38] Bhate A., Sun T., Li J. B. (2019). ADAR1: a new target for immuno-oncology therapy. *Molecular Cell*.

[39] Ishizuka J. J., Manguso R. T., Cheruiyot C. K. (2019). Loss of ADAR1 in tumours overcomes resistance to immune checkpoint blockade. *Nature*.

