# Supplementary figures and images for: The Interplay between RNA Editing Regulator ADAR1 and Immune Environment in Colorectal Cancer
Source: J Oncol. 2023 Jan 10;2023:9315027. doi: 10.1155/2023/9315027 (PMC9845036; doi:10.1155/2023/9315027)

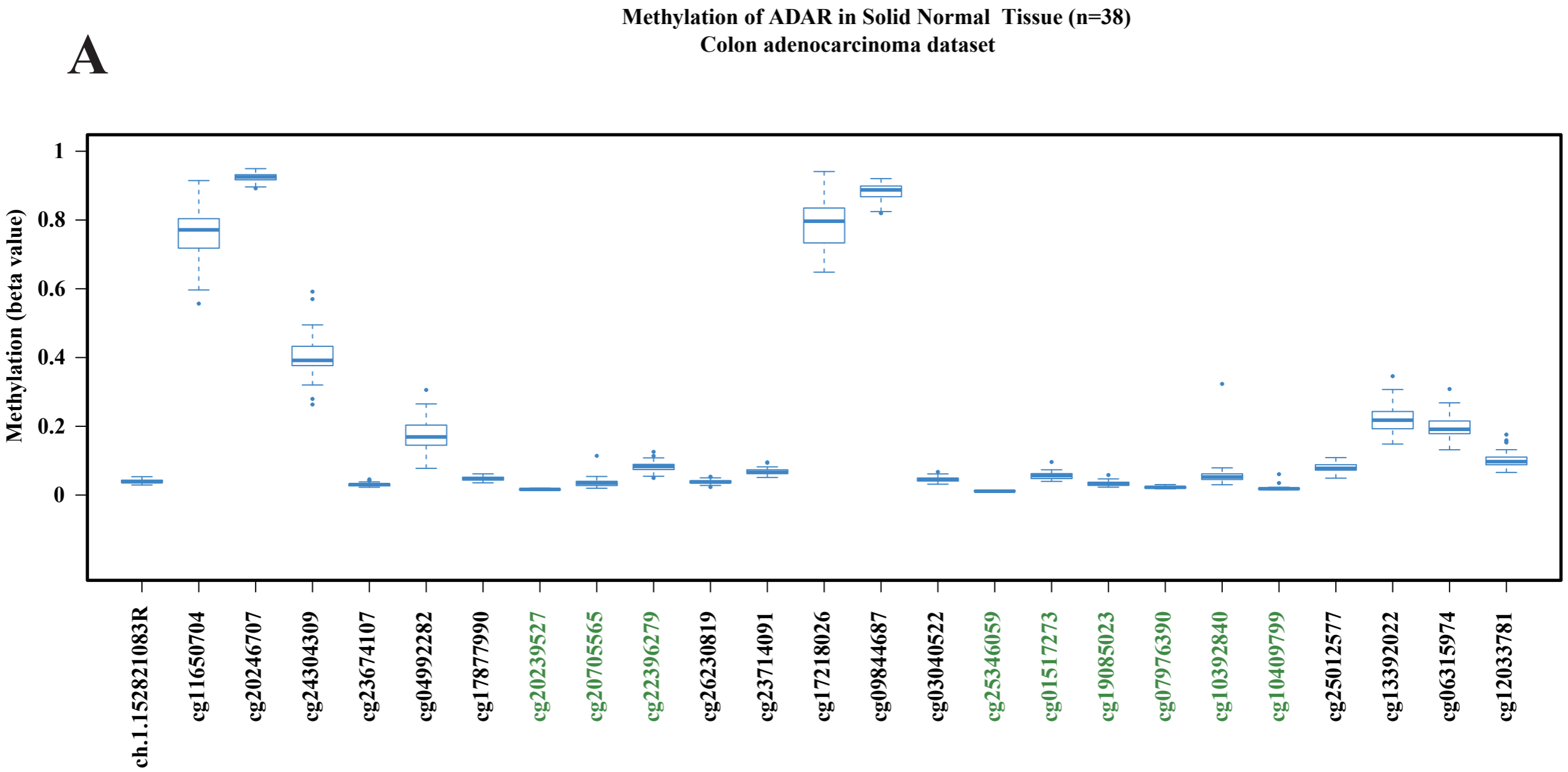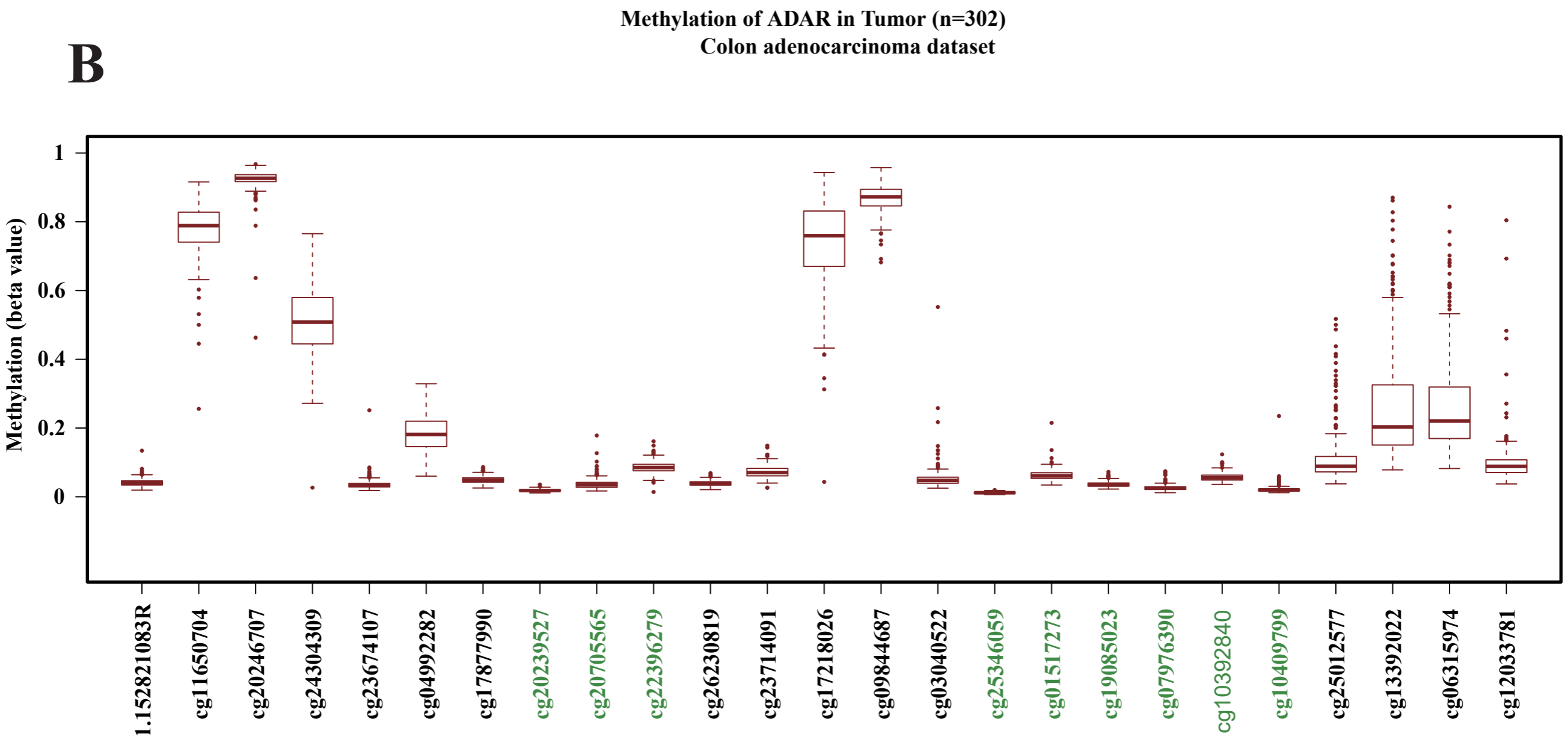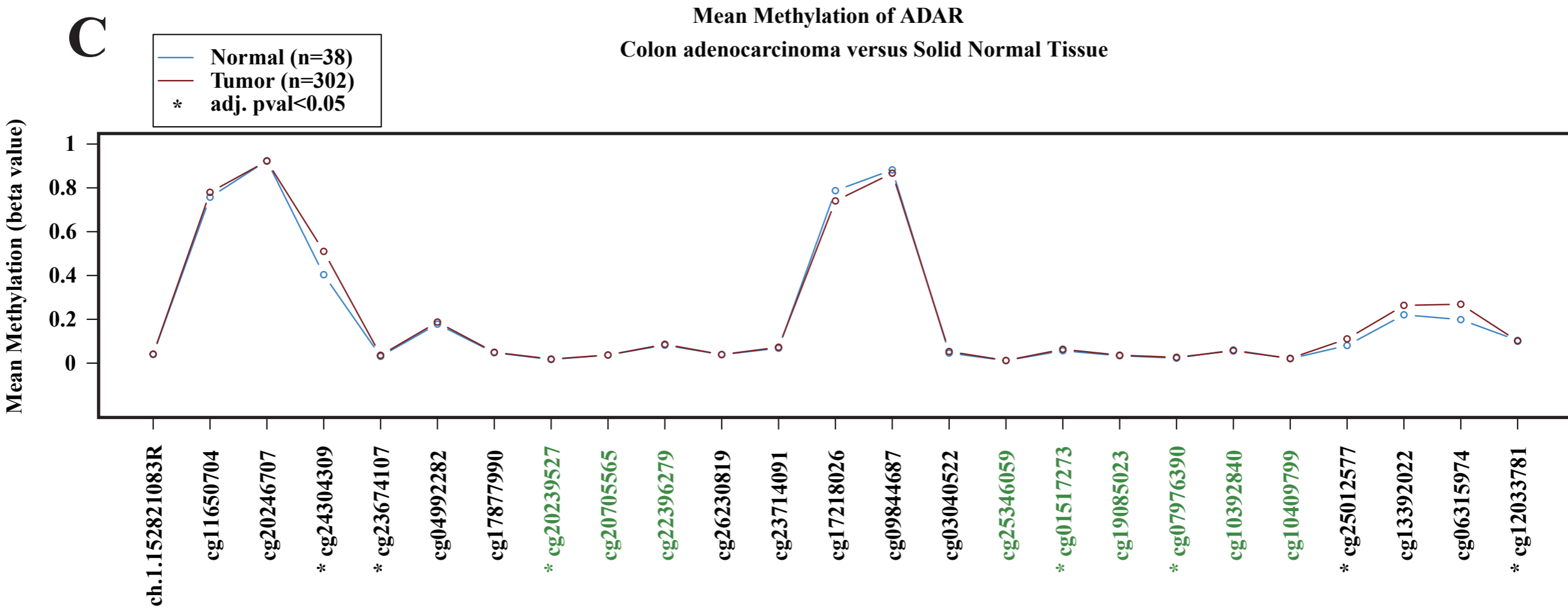

Supplement: Supplementary Materials — Supplementary Figure 1: ADAR gene-wide methylation based on Illumina Human Methylation 450 Bead Chip. (A) The Illumina 450K Methylation BeadChip methylation probes the distribution of the ADAR gene in solid normal tissues. (B) The Illumina 450K Methylation BeadChip methylation probes distribution of ADAR gene methylation in CRC tumor tissues. (C) The differential ADAR methylation probe between CRC and solid normal tissues. P < 0.05 was considered statistically significant. Data were obtained from the TCGA Wanderer database. [file 9315027.f1.pdf]
